# Context-informed incremental learning improves both the performance and resilience of myoelectric control

**DOI:** 10.1186/s12984-024-01355-4

**Published:** 2024-05-03

**Authors:** Evan Campbell, Ethan Eddy, Scott Bateman, Ulysse Côté-Allard, Erik Scheme

**Affiliations:** 1 Institute of Biomedical Engineering, University of new Brunswick, Dineen Dr., Fredericton, NB E3B 5A3 Canada; 2 Spectral Lab, University of New Brunswick, Peter Kelly Dr, Fredericton, NB E3B 5A1 Canada; 3 Department of Technology Systems, University of Oslo, Gunnar Randers vei, Kjeller, P.O Box 70 Norway

**Keywords:** Electromyography, Incremental learning, Active learning, Adaptation, Contextual learning, Myoelectric control

## Abstract

Despite its rich history of success in controlling powered prostheses and emerging commercial interests in ubiquitous computing, myoelectric control continues to suffer from a lack of robustness. In particular, EMG-based systems often degrade over prolonged use resulting in tedious recalibration sessions, user frustration, and device abandonment. Unsupervised adaptation is one proposed solution that updates a model’s parameters over time based on its own predictions during real-time use to maintain robustness without requiring additional user input or dedicated recalibration. However, these strategies can actually accelerate performance deterioration when they begin to classify (and thus adapt) incorrectly, defeating their own purpose. To overcome these limitations, we propose a novel adaptive learning strategy, Context-Informed Incremental Learning (CIIL), that leverages in situ context to better inform the prediction of pseudo-labels. In this work, we evaluate these CIIL strategies in an online target acquisition task for two use cases: (1) when there is a lack of training data and (2) when a drastic and enduring alteration in the input space has occurred. A total of 32 participants were evaluated across the two experiments. The results show that the CIIL strategies significantly outperform the current state-of-the-art unsupervised high-confidence adaptation and outperform models trained with the conventional screen-guided training approach, even after a 45-degree electrode shift (p < 0.05). Consequently, CIIL has substantial implications for the future of myoelectric control, potentially reducing the training burden while bolstering model robustness, and leading to improved real-time control.

## Introduction

Following decades of success in controlling powered prostheses [1], myoelectric control is becoming an increasingly sought-after hands-free input modality for human-computer interaction (HCI) [2]. Its convenience, subtlety, and intuitiveness make it a particularly attractive solution for emerging ubiquitous applications, such as in mixed-reality scenarios where camera-based approaches are infeasible. Leveraging the electromyogram (EMG) signals generated during muscular contractions and associating them with gestures enables hands-free, always-available input for various potential tasks. For example, myoelectric control has been used for human-robot interaction [3, 4], cursor control [5], sign language recognition [6], and more recently mixed reality [7]. Its primary use has been, and continues to be, prosthesis control, where it is commercially offered by several vendors (e.g., Coapt,^1^ Infinite Biomedical,^2^ and Ottobock^3^). However, despite its inherent promise, myoelectric control continues to suffer from several factors that hinder its real-world viability [8, 9].

A major impediment to the widespread viability of myoelectric control systems is their inherent susceptibility to degradation over time, influenced by factors such as electrode displacement, user fatigue, and shifts in muscular contractions [8]. Although systems can be recalibrated by acquiring guided training data, this process is tedious, especially for general-purpose applications where systems should be as close to “ready to use” as possible. Furthermore, the data collected during these controlled calibration sessions frequently fails to accurately reflect the patterns of contractions elicited during real-time device usage [10]. An ideal system would be capable of learning realistic user behaviours while maintaining the model’s robustness by continuously adapting to the user in real-time.

Unsupervised adaptation is one proposed solution that adapts to user-in-the-loop data without requiring direct user input, solving many of the issues associated with supervised recalibration [11]. Because it uses the classifier’s own outputs as pseudo-labels for adaptation, other fields have referred to this type of adaptation as *semi-supervised* [12]. This reliance on the classifier that is being adapted, however, leads to inherent limitations. For example, these approaches excel when preserving models that already perform well, producing pseudo-labels that are correct and confident. However, when faced with challenges such as a significant change in the input space—leading to reduced model accuracy—these unsupervised adaptation approaches may fail. As such, a crucial research objective is to combine the ground truth labels of supervised methods with the convenience offered by unsupervised methods.

To achieve the combined benefits of both methods, we introduce a novel approach called Context-Informed Incremental Learning (CIIL). This approach draws motivation from the demonstrated benefits of reinforcement learning in myoelectric control [13], while overcoming the user training burden introduced by the sample inefficiency of reinforcement learning approaches. Inspired by reinforcement learning paradigms, but remaining an unsupervised learning method, CIIL operates effectively in contexts where the user’s true intentions can be inferred. CIIL monitors the consequences of actions within an instrumented environment to assess their appropriateness, categorizing actions as positive or negative. Before adapting the model, the categories of actions provide an opportunity to reassign more appropriate labels if the classifier-assigned actions do not align with the inferred goal of the environment. Further, the context-informed labels can be used to isolate samples the model assigned an incorrect label (negative) or correct label (positive). This method overcomes the limitations of unsupervised adaptation strategies that often rely on the classifier’s confidence predictions being correct, as it focuses on the action’s suitability based on environmental context.

We evaluate the use of several CIIL strategies in two separate scenarios that could benefit from adaptation: (1) when limited training data is available and (2) when a drastic shift in the input space has occurred (i.e., after a 45-degree electrode shift). By extracting environmental context in a simple target acquisition game, we show how this additional context can not only maintain model performance over time but improve the underlying model behaviour compared to dedicated screen-guided training approaches. In a departure from conventional methods, our innovative CIIL training approach not only outperforms current standards but also holds substantial promise for advancing the effectiveness of next-generation myoelectric control systems.

## Related works

### Incremental learning in myoelectric control

The practice of collecting offline data (i.e., where contractions are elicited with no active control or feedback provided to the user) to train a classifier is arguably an incomplete solution when optimizing for online, user-in-the-loop interactions. Correspondingly, there is often weak association between offline classification performance and usability during later online use [10]. While other offline metrics may provide better predictive power [14], the best indicator of online usability is evaluating classifiers in more realistic intermediate tasks, such as a simple virtual environment [15]. This is, at least in part, because the performance of myoelectric control is dependent not only on the model’s fit but how well understood the user’s behaviour is under all circumstances (i.e., when a user’s mental bandwidth is split between adhering to consistent patterns as produced during training and performing their control task). As such, conventional guided calibration approaches, wherein a user mimics a sequence of prompted gestures used as labels for supervised learning (such as screen-guided, or prosthesis-guided, training [16]), prioritize the ability of the classifier to distinguish motion classes within the capacity of the controlled training dataset; but not necessarily for a more variable user-in-the-loop setting. Given the importance of capturing these user-in-the-loop behaviours for model performance, the online adaptation of model parameters during real-time user interaction is an appealing alternative to guided calibration techniques.

Traditionally, online adaptation has been explored as a mechanism to combat a decline in performance due to gradual physical and behavioural changes [17–19]. In the work done by Zhang et al. it was shown that unsupervised adaptation could improve model resilience to simulated random noise that was added to offline testing data [17]. More recently, it has been demonstrated that serious games provide an avenue for online adaptation to improve classifiers trained with screen-guided prompts [18, 20]. For example, Woodward et al. found that users elicited greater limb position variability during online use which, when accounted for through online adaptation, yielded significantly higher scores during functional tests for both intact-limbed and amputee populations. Furthermore, researchers have addressed performance decline in myoelectric control across days by developing recalibration strategies, such as the self-calibrating asynchronous domain adversarial neural network (SCADANN) introduced by Côté-Allard et al.. This approach effectively combines established myoelectric domain adversarial methods [21, 22] with stability-focused heuristics and, notably, demonstrated superior robustness through gradual refinement of its representation using minimal unlabeled data [19].

Nevertheless, most unsupervised adaptation research has assumed the availability of a reasonably robust initial model. Comparatively little work has explored its performance in light of a catastrophic concept shift (such as doffing/donning of the EMG recording device), when the previously trained classifier may yield ineffective pseudo-labels, or with little-to-no initial training. In such situations, the classifier’s predictions may result in incremental learning updates, reinforcing the same flawed representation or, worse, driving the model in unknown and irreproducible directions. Although auxiliary information, such as informativeness [23], has been used alongside classifier confidence to determine valid samples to reincorporate, this may prevent further degradation of a flawed model but does not enable recovery or improvement. Correspondingly, there is a need for adaptive techniques that are less susceptible to such factors.

**Fig. 1:**
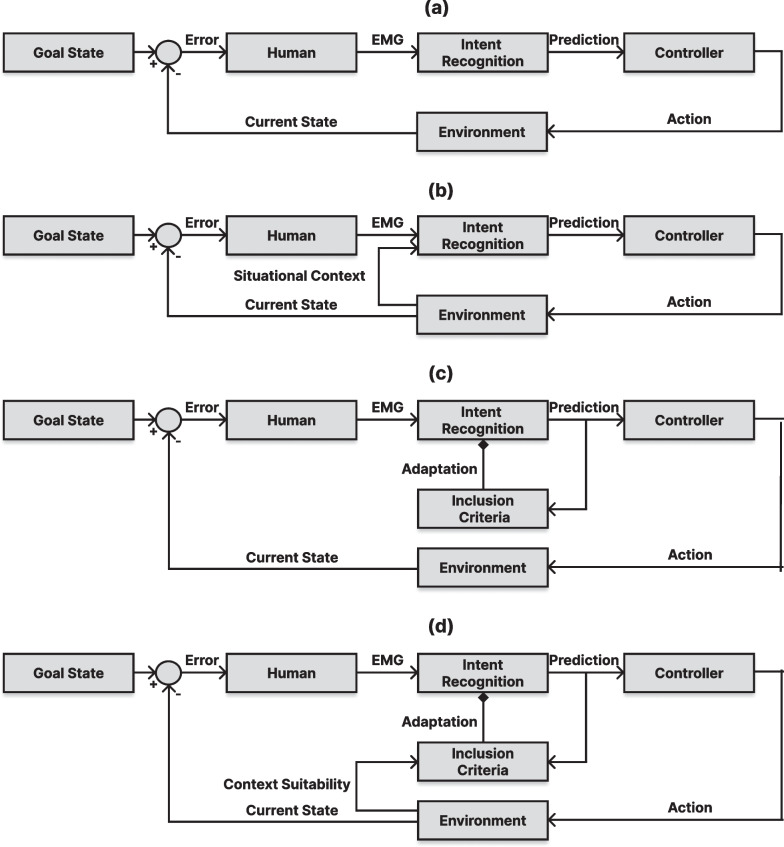
Depictions of various myoelectric control system designs. Solid lines indicate an output from a node or an input to a node, and a diamond indicates a signal that modifies the weights of a node.** a** Myoelectric control systems designed using traditional practices (optimized for open-loop control, user perception closes the loop).** b** Myoelectric control systems that are context-aware (CA) and modulate the intent relayed to the controller subject to environmental information.** c** Myoelectric control systems optimized via traditional incremental learning (IL) (these systems rely on the high confidence predictions of the classifier to incorporate incoming data to the adaptation set).** d** Myoelectric control systems that are supplied contextual feedback based on the suitability of the performed action in the environment and use this information to govern incremental learning (CIIL)

### Reinforcement learning

Unlike traditional supervised strategies that utilize pairs of inputs and labels, reinforcement learning uses a reward signal derived from the advantageous or disadvantageous outcomes of an agent’s interactions. This reward signal serves as a *direct* measure of the agent’s functional ability and can guide model updates independent of the classifier’s accuracy in predicting the user’s intent. Reinforcement learning has been explored in the myoelectric literature, although primarily for offline supervised strategies. Wu et al. demonstrated the effective learning of wrist and finger joint angles using proximal policy optimization, circumventing the drift typically encountered with supervised regression and forward dynamical simulations [24]. They defined the discrepancy between predicted and measured joint trajectories as a continuous-valued reward signal. Similarly, Vasconez et al. utilized deep Q-learning for gesture recognition, employing positive rewards for correct predictions and negative rewards for incorrect predictions based on data collected from screen-guided training [25]. Likewise, Edwards et al. used generalized value functions with EMG signals to elicit a switch command that changed the degree of freedom being controlled, a mechanism typically used alongside direct sequentially controlled prostheses [26]. Although these approaches showcase the application of reinforcement learning in myoelectric control, the reward signals utilized were not direct measurements of performance in functional tasks.

There are relatively few studies that explore reinforcement learning for myoelectric control with a reward signal that directly quantifies functional performance from a user-in-the-loop setting. Pilarski et al. has demonstrated that actor critic reinforcement learning can optimize the control of a robotic arm from reward signals that quantify functional performance in two ways [13]. The first method uses a binary success metric, issuing a reward of +1 when within a region around the target trajectory and a reward of $-$0.5 when outside this region. The second method was making the user directly assign the rewards using arrow keys during use, as was pioneered by the TAMER approach [27]. Using both approaches, the user could precisely control two joints of the robotic limb to the desired target after sufficient training (<25 min and <10 min for the binary and human-assigned reward signals, respectively). Despite the success of this approach, reinforcement learning approaches are notoriously sample inefficient [28]. This relatively rapid training time was achieved by first developing an efficient EMG input representation and further tile coding the inputs of the continuous values into discrete bins. This compressed representation may not extend to more rich myoelectric control tasks when greater spatial and temporal resolution are needed to decipher user intent. As such, there is motivation to learn how to harness such contextual information, but to leverage it in a more sample efficient way.

### Context

As characterized in human-computer interaction research, context is “any information that can be used to characterize a situation or entity” [29, 30]. By leveraging this often simple additional information, naive computer systems can become contextually aware, significantly improving the potential bandwidth of the particular human-computer input [31, 32]. For example, systems that leverage past input history as context can provide improved and tailored recommendations to users [33]. Alternatively, a user’s location could be used as context to improve search results on the web [34]. In turn, these contextually aware systems have become an expectation for many users, reducing the need for user input to enhance the user experience.

Despite its history of success within HCI, the concept of context has only recently begun to surface in prosthetics. For example, some have used decision stream context like majority voting [35] and rejection [36] to improve control. Similarly, Sensinger et al. proposed a form of decision stream context during unsupervised adaptation by not adapting to changes in contractions that were not physically possible (i.e., they happened too quickly) [11]. While both approaches utilize the temporal context of the decision stream, they cannot be considered fully contextually aware systems. This is because their reliance on a potentially incorrect underlying model may result in a context that is isolated from, and possibly in disagreement with, the real-world environment. Others have sought to improve situational context by including additional sensors, such as cameras embedded within the prosthesis [37–39]. These are examples of context-aware myoelectric control systems that incorporate environmental information into their classifier inputs (see Fig. 1b). Although such systems can greatly improve control by providing additional information to the underlying classifier [38, 40], they do not attempt to correct the model’s behaviour. Moreover, these techniques may require the introduction of additional sensors, hardware, or environmental constraints and may reduce the user’s agency over the system—thus leading to abandonment of the device [41].

In this work, we lean on context to better derive pseudo-labels during unsupervised adaptation. In this novel paradigm, the context from the environment (i.e., the task’s setting) acts as a supervisory layer to inform the active learning process and correct the underlying model rather than the output. This becomes particularly interesting for general-purpose myoelectric settings where context becomes more trivial to extract. For example, environmental context could be a successful or missed button click. Alternatively, context could be quick error corrections such as quickly opening and closing a menu in mixed reality. By leveraging this seemingly simple context to continuously refine and enhance a model’s performance, the viability of myoelectric control incrementally improves, encompassing not only prosthetic control but also extending to mixed reality environments where the context might be even more immediately perceptible.

## Methods

### A proxy environment for online adaptation

This work employs a simple target acquisition game to provide a context-aware environment from which real-time adaptation can be performed (see Fig. 1b). In this game, participants controlled a crosshair along two independent degrees of freedom (Left/Right and Up/Down) with the goal of “saving” as many planets as possible in the given amount of time. After successfully moving the cursor over the top of a planet and hovering within its bounds for three seconds, the planet spawned at a different location. This cycle continued for the game’s duration. To keep consistency across participants and trials, the sequence of positions in which the planets were generated remained the same for all participants and in each trial. Although contrived, this environment serves as a proxy for any myoelectric control setting where context can be inferred.

**Fig. 2:**
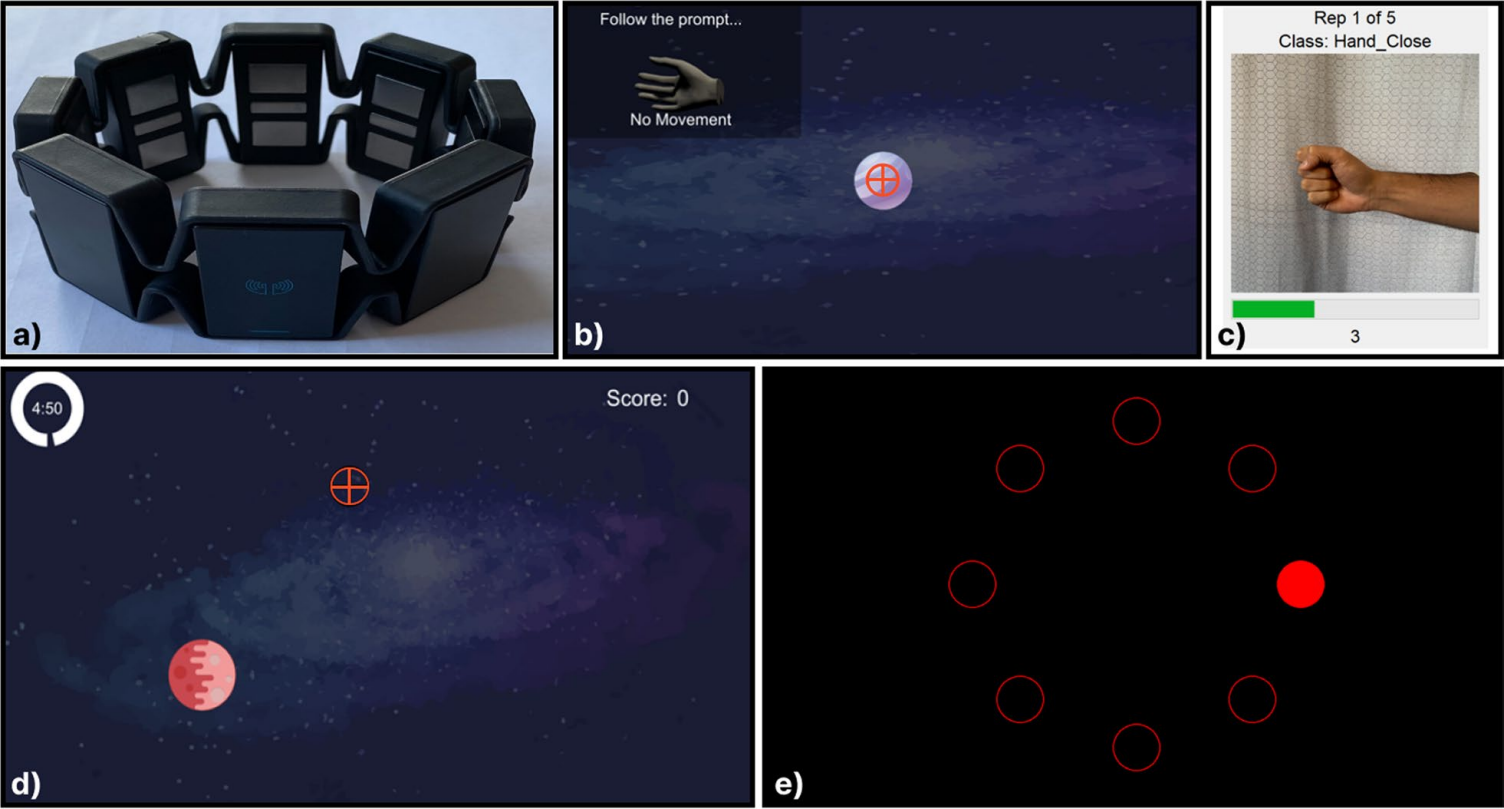
Experimental setup used throughout the study.** a** The Thalmic Labs Myo.** b** The calibration screen used in Scenario 1 collects one second of EMG data per motion class.** c** The screen-guided training interface for collecting offline data.** d** The gamified proxy environment, Myoshoot, where adaptation occurred. The user’s position was represented by a crosshair and the goal was represented by a planet.** e** The Fitts’ law environment where online usability was ultimately tested

### Control scheme

The Myo Armband, a previously commercially available dry-electrode EMG cuff that records data at 200 Hz across eight channels, was used in this work. To discriminate between inputs, data were split into windows of 200 ms with 100 ms increments corresponding to 40 samples per window with an increment of 20 samples between windows. Hudgins’ time domain features were extracted from each window [42]. A linear discriminant analysis (LDA) classifier was then used to differentiate between the five gestural commands (Wrist Flexion—Left, Wrist Extension—Right, Hand Open—Up, Hand Close—Down, Neutral Gesture—No Motion). After training, the LDA model was adapted in real-time during the aforementioned target-acquisition game using ten-second batches of data. This interval was chosen empirically through pilot testing and was a trade-off between user adaptation to the changing control scheme and optimal adaptation performance [43]. The mean and covariance of each class were computed and combined with the existing LDA parameters using a static adaptation rate of 0.1 and a scaling factor reflecting the effective sampling rate. The adaptation of the mean and covariance matrices is shown below in equations (1) and (2):

1 $$\begin{aligned} a= & {} \frac{ \alpha N_{b,c}}{N_{c}+\alpha N_{b,c}} \end{aligned}$$

2 $$\begin{aligned} \mu _{c,i}= & {} (1-a) \mu _{c,i-1} + a\hat{\mu _{c}} \end{aligned}$$

where $\mu _{c,i}$ is the mean of class *c* after adaptation, $\mu _{c,i-1}$ is the current mean of class *c*, $\hat{\mu _{c}}$ is the mean of class *c* of the batch, *a* is the adaptation rate, $\alpha$ is the static adaptation rate, $N_{b,c}$ is the number of new samples of class *c*, and $N_c$ is the total number of samples in class *c* collected for adaptation thus far.

* Note that this work was built with LibEMG, an open-source Python library for designing and evaluating real-time myoelectric control systems [44]. The code and data can be found at *github.com/ECEEvanCampbell/CIIL_LDA*.

### Incremental learning strategies

**Fig. 3:**
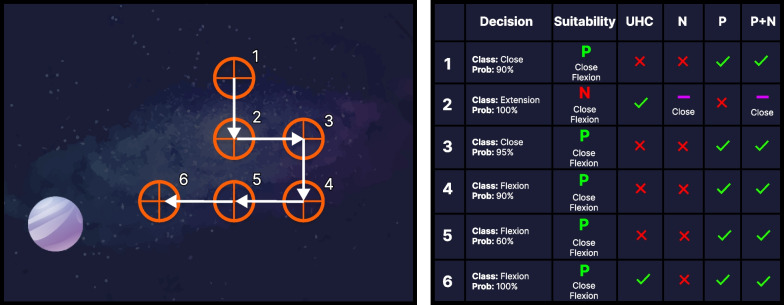
A representation of a hypothetical sequence of cursor movements in the control task. The crosshair indicated the cursor position at each timestep with arrows indicating the direction of travel for the next timestep. An interpretation of the steps along the trajectory is given in the right table, where the decision (class and probability) and context suitability (was the performed action correct (P or N for positive or negative) and what were the correct options under the context) served as inputs to the inclusion criteria of different algorithms. The effects of the different algorithms are given by checkmarks (include with classifier’s prediction), hyphens (override the decision using context), and x’s (discard the sample)

To improve the usability of myoelectric control systems beyond the current status quo, we introduce the concept of **Context-Informed Incremental Learning (CIIL)**. In the case of myoelectric control, context can be acquired through task-specific constraints, knowledge, or additional sensors. The system then gets *feedback* based on its perceived contextually-defined performance, and using this feedback, the model can *learn* from its actions and make improvements (see Fig. 1d). The model is then *incrementally* updated based on the contextual knowledge acquired, leading to more robust control systems that are defined by active in situ user behaviour. This work highlights three categories of CIIL systems (Negative, Positive, and Positive + Negative) and compares their performance to the state of the art adaptive system (unsupervised high-confidence). An overview of the these strategies is presented in Fig. 3.

#### **Negative context-informed incremental learning (N-CIIL)**

Attempts to correct incorrect (i.e., negative) model behaviour by updating the predicted label with the assumed “correct” one. In the target-acquisition environment, incorrect actions were flagged when the cursor moved away from the target, indicating a disagreement between the task objectives and user behaviour. As the cursor could only be controlled along one axis at a time, two directions could usually be deemed correct (i.e., the diagonal between the cursor and the target). The training cluster whose mean was closest in Euclidean distance to the newly elicited contraction was selected to differentiate between the two potentially correct labels. Note that all positive actions were ignored and not used to adapt the system.

#### **Positive context-informed incremental learning (P-CIIL)**

Reinforces positive model behaviour by adapting the model with predictions that lead to positive within-task results. In the target-acquisition environment, positive actions were flagged when the cursor moved toward the target, indicating that the user was accomplishing the task objectives. Correspondingly, the assumption was that the model made a correct decision that should be reinforced. All negative actions were ignored and not used to adapt the system.

#### **Positive + negative context-informed incremental learning (P+N-CIIL)**

A combination of positive and negative CIIL where all of the model’s predictions are leveraged for adaptation. Positive outcomes are adapted using P-CIIL, and negative outcomes are adapted using N-CIIL.

#### **Unsupervised high-confidence (UHC)**

A popular extension of unsupervised adaptation where a model is updated with all decisions that have a confidence value above some predefined threshold [11]. Although this strategy uses the additional context of the classifier’s confidence profile, it is not considered a CIIL strategy as it does not leverage context beyond the knowledge of the underlying model.

### Outcome measures

#### Offline metrics

Three offline performance metrics were leveraged to assess the performance of each model: classification accuracy, active error, and instability [45]. Accuracy is a common metric that captures the number of correct predictions as a percentage of the total predictions. In contrast, active error is a specialized metric that focuses only on misclassifications that resulted in unwanted movement, and does not penalize misclassifications that were predicted as no motion. Instability is a metric that captures the number of class transitions seen in the decision stream. If analyzing data obtained through screen-guided-training, which typically does not record class transitions, the observed instability should ideally be zero. These metrics were computed on the screen guided training data acquired during each experiment.

#### Online metrics

To evaluate the online performance of each adapted model, an ISO 9241-9 inspired Fitts’ Law test evaluation (see Fig. 2c) was used. Fitts’ Law is broadly used for human-computer interaction studies and provides an avenue for testing pointing and selection tasks [46]. During this online usability test, participants were required to capture a set of eight targets arranged in a circle. Although similar to the target acquisition game, no model adaptation occurred during this task. Additionally, participants were told they could give up at any time if they felt they did not have the control required to complete the test. Three commonly used online metrics were assessed during this task: throughput, path efficiency, and overshoots [47]. Path efficiency is the ratio of the shortest possible path from the cursor to the target to the actual path taken. The overshoot metric—the number of times the cursor enters and leaves a target before acquisition—is particularly important for measuring the stability of the control scheme during target acquisition. Finally, the throughput of the system is a measurement of the overall performance and is computed by the following equations:

3 $$\begin{aligned} TP= & {} \frac{1}{T}\sum _{i=1}^{T}\frac{ID_{i}}{MT_{i}} \end{aligned}$$

4 $$\begin{aligned} ID= & {} \text {log}_{2}\left( \frac{D}{W} + 1\right) \end{aligned}$$

where *T* is the number of trials, *ID* is the index of difficulty, *MT* is the movement time, *D* is the distance to the target, *W* is the width of the target, and *i* is the trial number.

## Experimental design

In this work, we evaluated the CIIL strategies during two situations that could benefit from adaptation: (1) when there is minimal training data (e.g., when adapting to a new user) and (2) when the input space has catastrophically shifted (e.g., after a 45 degree electrode shift). A total of 32 participants took part in these online, user-in-the-loop experiments, as described in the following sections. At the start of the session, participants were asked to place the EMG cuff in a comfortable position on their right forearm. After giving the armband time to adjust to the participants’ skin and humidity (approximately 5 min), the initial model (i.e., the baseline for all the adaptation trials) was trained. The participants then went through a progression of in-game adaptation on the baseline classifier, proceeded by an ISO Fitts’ evaluation of the adapted model. Additionally, two repetitions of steady-state, open-loop (no feedback) contractions were collected through screen guided training after each cycle to acquire a representative testing sample. This proceeded until all the adaptation strategies had been tested. All participants gave written informed consent prior to beginning, as approved by the University of New Brunswick’s Research Ethics Board and are on file as REB (2022-122).

### Experiment scenario 1: minimal training data

Experiment one consisted of a pilot study to evaluate the efficacy of the CIIL strategies on a sparse yet reasonably accurate starting classifier. The preliminary results of this study were described in [48]. Eleven participants were recruited for this initial work (4 female, 7 male; ages 20–56). To train the starting model, one second of training data for each of the five classes using the built-in data collection screen (see Fig. 1A) were acquired. The goal was to train a model whose feature space was correct but sparsely populated, possibly leading to unstable classifier behaviour. Although this limited amount of data is known to be suboptimal, commercial EMG systems have promoted the use of single repetition calibration protocols (e.g. Myo) to prioritize an expedited setup at the expense of performance. We also acquired five repetitions of steady-state contractions through screen guided training to evaluate the performance of a classifier trained on offline data (i.e., the optimal baseline). Correspondingly, the goal of the first experiment was an initial analysis of the newly proposed CIIL adaptation strategies for a relatively simple problem. The unique design constraints of the first experiment are as follows:

#### Velocity

To simplify the control system, we opted for simple constant-velocity control mapping meaning that the crosshair moved at a constant speed regardless of contraction intensity.

#### UHC confidence threshold

The confidence threshold was empirically tuned during pilot studies to give the best outcome for UHC. Because the concept drift between training and testing is small and the confidence distribution of LDA is skewed high [49], the threshold was set to 100%. This conservative confidence threshold of 100% was chosen to adapt the model in the most cautious way possible. Even at this threshold, approximately 50% of within-game decisions were used to adapt the model.

#### Negative CIIL

In addition to the mechanisms of N-CIIL described above, we assumed any movement within the acquired target to be unwarranted and thus used it as negative context.

#### Order

The trial order was randomized across participants in this within-participant study. The six unique trials included the use of the screen guided training model (SGT), the initial one second model (initial), and the four unsupervised strategies (UHC, P, N, P+N).

#### Gameplay time

The baseline model was adapted during a two-minute gameplay session—the approximate equivalent of acquiring five repetitions of offline SGT data.

### Experiment scenario 2: electrode shift

In a second, separate, phase of the study, the efficacy of the CIIL strategies was evaluated when a model’s input space had been catastrophically shifted. An additional 21 participants were recruited for this experiment (8 female, 13 male; ages 18–41). Participants trained an initial model using five repetitions of ramp contractions, increasing from rest to their desired activation level over three seconds, for each input class. Then, after acquiring the baseline performance of the model in an ISO Fitts’ test, participants were asked to take off and rotate the EMG cuff by 45 degrees clockwise from their perspective ($\sim$ one electrode). This simulated doffing and donning of the EMG cuff in a different location with the amount of shift likely being even more extreme than what might be reasonably expected in a prosthetic socket. While dealing with electrode shift through automatic approaches is an ongoing area of research [50], its role in this experiment was intended as a proxy for any time the input space has drastically shifted, such as after doffing and donning a device, between-day control, or even after cross-user adaptation [22]. The unique design constraints of the second experiment were as follows:

#### Velocity

A proportional control mapping was used, meaning harder contractions resulted in faster speeds. Subject’s class-specific mean absolute value was used to determine 20% and 70% thresholds that formed a linear mapping for 0% to 100% of the available cursor speed, as per [51].

#### UHC confidence threshold

Given that the concept drift introduced by the electrode shift was large, a lower confidence threshold of 99% was found to be best for UHC from pilot studies. This selection allowed UHC to include some incoming samples to attempt to improve; whereas too few samples would have been gathered with a threshold of 100%.

#### Negative CIIL

The negative adaptation from the first experiment was modified so that only within-target movement that drifted away from the center was considered as negative context.

#### Order

The order of the trials (P, N, P+N, UHC) was balanced across participants using a Latin square. Each user started with the pre-shifted SGT model.

#### Gameplay time

To observe a plateau in the adapted model that wasn’t seen with the two minutes of adaptation from experiment one, an adaptation time of five minutes was selected.

#### Questionnaire

Participants completed a NASA TLX survey after the ISO Fitts’ test to better gauge the perceived workload of controlling the cursor with each adapted model. The questionnaire queried the quality of the models *after* adaptation had occurred, and does not reflect the user’s perception of the model during the gameplay session.

### Statistical analyses

Statistical analyses were conducted using the Statistical Tests for Algorithms Comparison (STAC) platform [52]. Analyses were performed independently for each experiment to determine differences between learning strategies from observed offline metrics (classification accuracy, active error, instability) and online metrics (overshoots, path efficiency, throughput). For each metric, normality and homogeneity of variance were tested and Friedman ranking tests were determined to be appropriate.

Following a significant outcome, post-hoc multiple comparison tests were conducted using Finner correction [53] with a confidence level of 0.05. The resulting multiple comparison tests were visualized with critical difference diagrams. Within these diagrams, the mean normalized rank of the pipelines were determined and plotted on a number line. The lowest ranked pipeline is indicative of the lowest values among the pipelines for the metric being analyzed, whereas the highest rank is indicative of the highest values among the pipelines for the metric being analyzed. As such, the most desirable (best performing) rank for the metrics analyzed in the study were highest for accuracy, lowest for active error, lowest for instability, lowest for overshoots, highest for efficiency, and highest for throughput. Finally, these diagrams also illustrate when pipelines are not significantly different by grouping them together with a horizontal bar.

**Fig. 4:**
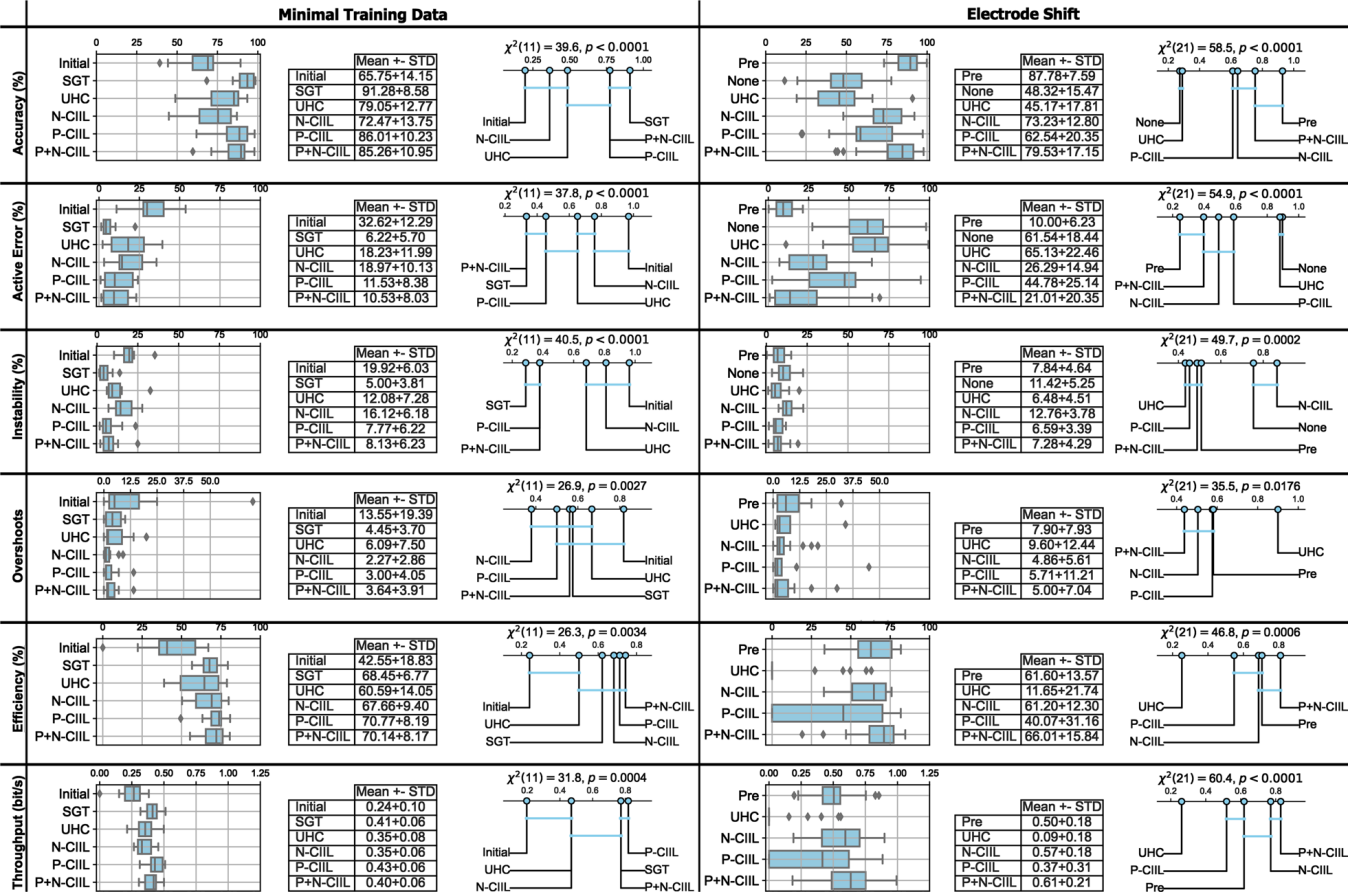
Box plots, summary statistics, and critical difference diagrams for offline metrics (accuracy, active error, instability), and online metrics (overshoots, efficiency, throughput) in both evaluated scenarios (minimal training data, electrode shift). Ideal trends for values and normalized ranks of these metrics are high accuracy, low active error, low instability, low overshoots, high efficiency, and high throughput. Blue bars linking groups in the critical difference diagrams signifies a non-significant difference within the multiple comparison tests. Note: when subjects did not recover from the introduced electrode shift and could not complete the online assessment, their data was removed for the overshoots metric as a placeholder value was not appropriate

## Results

The quantitative outcomes of scenario 1 were compiled into the first column of Fig. 4.For offline metrics, the SGT model was found to have significantly higher classification accuracy than the initial model, N-CIIL, and UHC adaptation (91.28% vs. 65.75%, 72.47%, and 79.05%, respectively; $p<0.05$), but not P+N-CIIL or P-CIIL (85.26% and 86.01%). For active error, the P+N-CIIL and SGT models significantly lower than the initial model, N-CIIL, and UHC (10.53% and 6.22% vs. 32.62%, 18.97%, and 18.23%, respectively; $p<0.05$), but not P-CIIL (11.53%). Likewise, instability was significantly lower for SGT, P-CIIL, and P+N-CIIL compared to the initial model, N-CIIL, and UHC (5.00%, 7.77%, and 8.13% vs. 19.92%, 16.12%, and 12.08%, respectively; $p<0.05$). For the online metrics, N-CIIL had significantly lower overshoots compared to the initial model (2.27 vs. 13.55; $p<0.05$), but not P-CIIL, P+N-CIIL, SGT, and UHC (3.00, 3.64, 4.45, and 6.09, respectively). Path efficiency of SGT, N-CIIL, P-CIIL, and P+N-CIIL was significantly higher than the initial model (68.45%, 67.66%, 70.77%, and 70.14% vs. 42.55%, respectively; $p<0.05$). Finally, the throughput of P-CIIL was significantly higher than the initial model, UHC, and N-CIIL (0.43 bit/s vs. 0.24 bit/s, 0.35 bit/s, and 0.35 bit/s, respectively; $p<0.05$), but not SGT or P+N-CIIL (0.41 bit/s and 0.40 bit/s).

The quantitative outcomes for scenario 2 were compiled into the second column of Figure 4. For offline metrics, the pre-shift scenario had significantly higher classification accuracy than the shifted scenario with no adaptation, UHC, P-CIIL, and N-CIIL (87.78% vs. 48.32%, 45.17%, 62.54%, and 73.23%, respectively; $p<0.05$), but no difference was found with P+N-CIIL (79.53%). The active error multiple comparison had similar outcomes, with the pre-shifted setting significantly outperforming the shifted scenario with no adaptation, UHC, P-CIIL, and N-CIIL (10.00% vs. 61.54%, 65.13%, 44.78%, and 26.29%, respectively; $p<0.05$), but not P+N-CIIL (21.01%). As a consequence of not recovering all gestures during adaptation for most subjects, UHC had the lowest instability (manifested by failing to elicit all classes); however, no significant differences were found between UHC, P-CIIL, P+N-CIIL, and the pre-shifted setting (6.48%, 6.59%, 7.28%, and 7.84%, respectively)—of which P+N-CIIL and the pre-shifted setting could elicit all classes for all subjects. For the online metrics, UHC had significantly more overshoots than the pre-shifted setting, P-CIIL, N-CIIL, and P+N-CIIL (9.60 vs. 7.90, 5.71, 4.86, and 5.00, respectively; $p<0.05$). Path efficiency of P+N-CIIL was significantly better than P-CIIL and UHC (66.01% vs. 40.07% and 11.65%, respectively; $p<0.05$), and was marginally better than the pre-shifted setting and N-CIIL (61.60% and 61.20%, respectively). Finally and most importantly, the throughput of P+N-CIIL was significantly higher than all other methods including the pre-shifted setting (0.61 bit/s vs. 0.50 bit/s; $p<0.05$), but was only marginally higher than N-CIIL (0.57 bit/s).

**Fig. 5:**
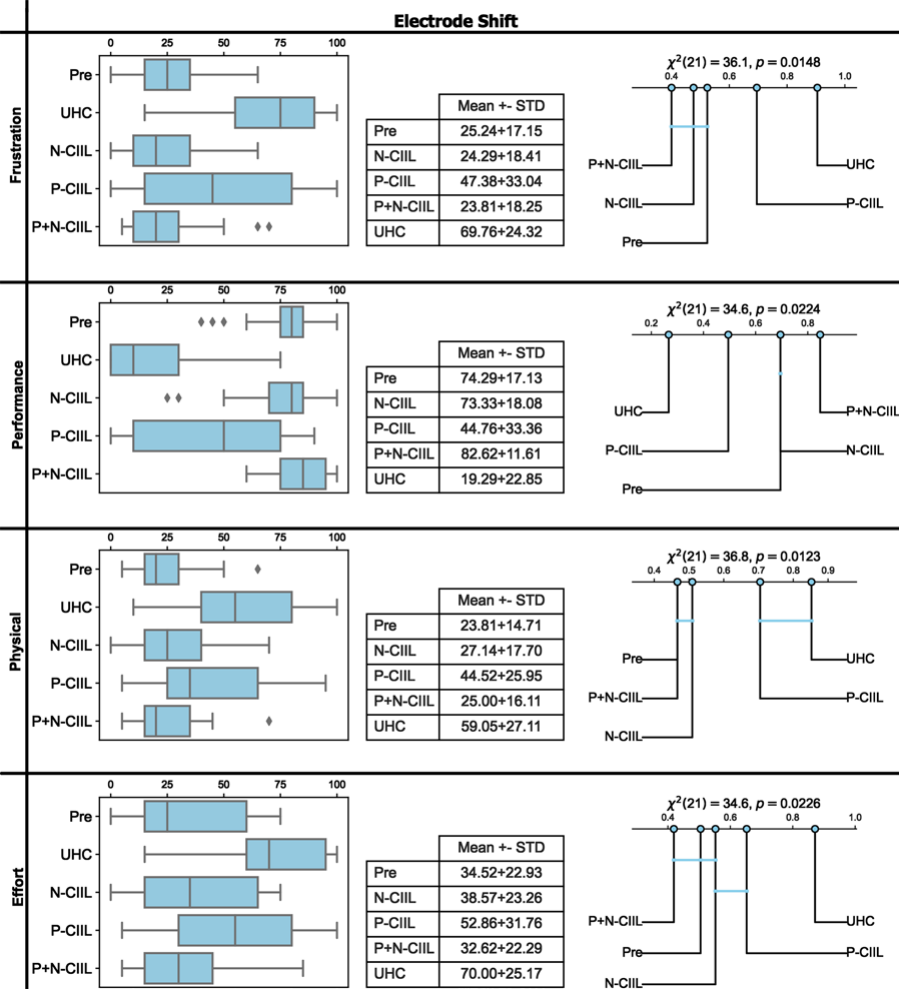
Box plots, summary statistics, and critical difference diagrams for subjective ratings of the different adaptation methods (frustrating, performance, physical effort, and overall effort ). Ideal ratings and normalized ranks for these categories are low frustration, high performance, low physical demand, and low overall effort. Blue bars linking groups in the critical difference diagrams signifies a non-significant difference between linked strategies

The qualitative outcomes for scenario 2, which were extracted from the NASA TLX survey, are shown in Fig. 5. Reported frustration levels were lowest for P+N-CIIL, N-CIIL, and the pre-shift model with the P-CIIL and UHC models being significantly higher (23.81, 24.29, and 25.24 vs. 47.38 and 69.76, respectively; $p<0.05$). In addition to P+N-CIIL having the highest throughput (objective performance measure), P+N-CIIL was also reported to be the significantly best-performing model (subjectively perceived performance measure, 82.62). The pre-shifted SGT, P+N-CIIL, and N-CIIL models had significantly lower physical demand than the P-CIIL and UHC models (23.81, 25.00, and 27.14 vs. 44.52 and 59.05, respectively; $p<0.05$). Likewise, P+N-CIIL and the pre-screening SGT model had significantly lower overall effort compared to P-CIIL and UHC (32.62 and 34.52 vs. 52.86 and 70.00, respectively; $p<0.05$), but not N-CIIL (38.57).

## Discussion

### Adaptation dynamics

In evaluating the various myoelectric control adaptation strategies across the two experiments of the study, clear differences between approaches emerged.

Unsupervised high-confidence adaptation—the current standard for unsupervised learning—was beneficial when the system was already performing well, as demonstrated in the minimal training data experiment (i.e., Experiment 1). Because the class distributions were sufficiently informed, the UHC approach was able to continue to refine them appropriately. However, it does not yield any measurable benefits when the initial model is underperforming and would benefit the most from adaptation. Within the second experiment, only 5 of 21 subjects had sufficient recovery after the electrode shift to complete the usability test, and even in these cases throughput after UHC adaptation was drastically lower than the CIIL approaches. The failure of UHC to recover after catastrophic shift is a crucial drawback for its viability to maintain system robustness for myoelectric control where these perturbations are common. Nevertheless, UHC does not have the same contextual reliance as the other approaches, highlighting its usefulness and potential practicality for maintaining already robust models with minimal overhead when contextual information in unavailable.

P-CIIL demonstrated similarities with UHC, allowing the model to enhance its performance following successful actions. However, P adaptation solely focuses on positive outcomes, ignoring negative actions which are most commonly the result of misclassifications. It achieved significantly higher throughput than UHC when the initial model was reasonable (Experiment 1), and was more reliable than UHC when the initial model was performing poorly (Experiment 2). Its shortcomings primarily emerge when the classifier aligns with a ‘correct’ contextual direction that was not intended by the user. In the environment used in this study, this could arise when the planet is diagonal from the crosshair (up and left), the user tries to go left but the classifier went up. In this scenario, P-CIIL ends up reinforcing this inadvertent direction, albeit less frequently than UHC thanks to the reliance of contextual suitability of the direction. P-CIIL also runs into difficulties when the user was completely unable to evoke any positive context (for example when they get stuck in a certain mode). Consequently, only 14 of 21 subjects were able to recover after the electrode shift to a high throughput model when relying on positive context alone.

N-CIIL offers a contrasting approach to P-CIIL by enabling model recovery in the presence of mistakes, regardless of classifier performance. Even when employing a simplistic tie-breaking system dependent on the classifier’s best guess among the valid options, N-CIIL consistently demonstrated recovery capabilities, as evident in the electrode shift experiment. This holds true, even though the adaptation process had to choose between two potential behaviors during adaptation, inevitably leading to erroneous information being provided at times. Consequently, every subject was able to recover after the electrode shift to a usable state (i.e., they could complete the online Fitts’ law task). However, in longer adaptation scenarios, its performance may plateau as errors decrease in frequency, leading to a weakened adaptation signal.

**Fig. 6:**
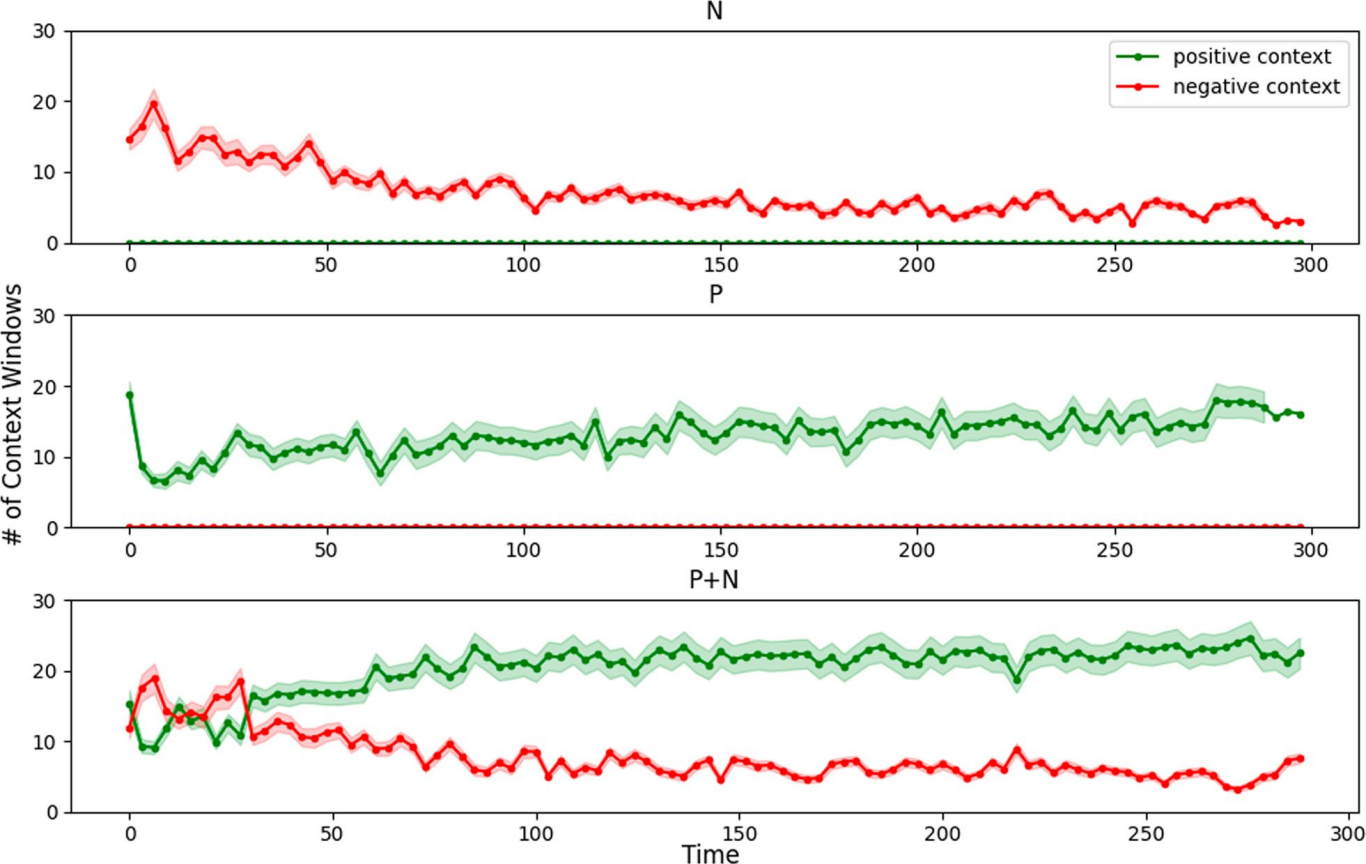
Representation of the number of context windows used by the CIIL pipelines the electrode shift scenario. Standard error across subjects is shown by a shaded region. Each point represents a three second time frame

A hybrid approach, combining positive and negative adaptation (P+N-CIIL), capitalizes on mistakes in the early stages and leverages positive interactions as they emerge. Generally, N-CIIL can recover a completely flawed model to the point where it can then benefit from P-CIIL. In doing so, this strategy harnesses the strengths of both adaptation approaches and exhibits the highest throughput in both experiments. This shift from negative to positive context for the P+N-CIIL pipeline is shown in Fig. 6, where P+N-CIIL began by using more negative context to accommodate the challenging setting, before transitioning to predominantly positive context after initial improvements. P+N-CIIL not only recovered to a usable level for every subject, but achieved a **significantly higher throughput to the pre-shift screen guided training model**. These findings underscore the significance of selecting an appropriate adaptation strategy based on the specific performance context and objectives in myoelectric control systems

### Qualitative feedback

In addition to quantitative measures of performance (throughput) being better post-shift post-adaptation in Scenario 2, self-reported qualitative measures of performance (extracted from the NASA TLX) showed users preferred the P+N-CIIL adapted model over the pre-shift SGT model. From the reported performance metric, P+N-CIIL was significantly better than all other models. Further, P+N-CIIL marginally outperformed the pre-shift SGT model in regards to having lower perceived effort and lower frustration. Overall, these qualitative measures corroborate the quantitative results, further supporting P+N-CIIL as the superior option.

### Offline vs. online performance

Offline accuracy is often used as an indicator for anticipated model performance during user-in-the-loop settings, however, the associative relationship between these two metrics is often debated. As highlighted in "Incremental Learning in Myoelectric Control" section, offline accuracy has a weak associative relationship to throughput and therefore should not be used to decide what algorithm would result in the best online control [10]. Recently, Hinson et al. demonstrated that offline metrics do correlate, albeit on a granular level, with online performance by training EMG to kinematics models to different levels of described variances ($R^2=$0.8, 0.6, 0.4) and performing Fitts’ law tests [54]. Hinson suggests the reason for the stronger relationship was due to experimental challenges in other works (altered outputs from controller gain [55], inadequate task duration and feedback [56]).

**Fig. 7:**
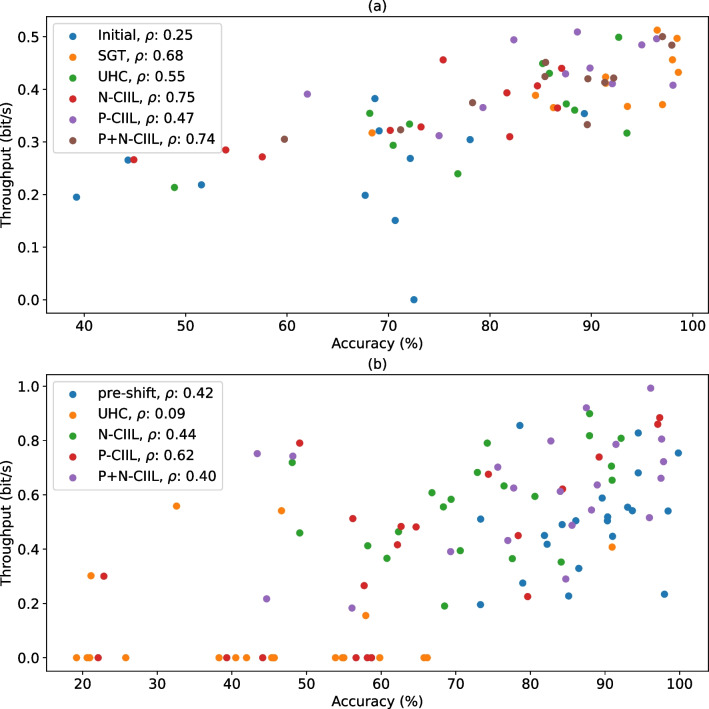
Scatterplot of associative relationships between offline accuracy and online throughput from** a** Scenario 1 (Minimal Training Data) and** b** Scenario 2 (Electrode Shift). Grouped Spearman’s correlation coefficients are presented in the legend. Global correlations, as calculated using all data irrespective of model, were $\rho = 0.74, 0.62$, for Scenario 1 and 2, respectively

Despite having intentionally introduced minimal data and electrode shift as experimental challenges in this study, we still observed “strong” correlations between offline accuracy and online throughput for both scenarios (global correlations of 0.74 and 0.62 for Scenarios 1 and 2, respectively, as shown in Fig. 7). Nevertheless, for the electrode shift scenario, the model with the highest accuracy differed from that with the highest throughput. In fact, although SGT produced the highest accuracy, P+N-CIIL significantly outperformed it in terms of throughput ($p < 0.05$). This discrepancy is justified given the behavioural differences a user exhibits while in-the-loop, but it illustrates that subtle differences in offline classification accuracy cannot be used naively to justify model rankings.

Moreover, classification accuracy computed on steady-state or ramp contractions may become irrelevant when it comes to incremental learning approaches such as those proposed in this work. For example, there were multiple cases where users unintentionally combined the hand open and closed contractions with radial and ulnar deviation, respectively, as they found this more intuitive to control the up and down directions of the cursor. While this could lead to improved within-task outcomes (i.e., increased throughput), it may also degrade the classification accuracy. Correspondingly, this tradeoff between these two metrics must be considered as we continue to move toward adaptive systems, and a particular emphasis should be placed on how to best evaluate these systems.

### Practicality of CIIL

While this study offers an important initial demonstration and validation of CIIL, the evaluation environment was relatively simple compared to the diverse settings where myoelectric control might be applied, such as dexterous manipulations of prosthetic limbs or mixed reality scenarios. Nevertheless, it is worth emphasizing that the potential benefits of CIIL may be most pronounced in these more complex and dynamic settings, if situational context can be appropriately measured or inferred.

In prosthetics, determining the suitability of control actions would require knowledge of appropriate gestures given the task being conducted. For example, near field communication tags could be used in the home to notify the device of an appropriate gesture when reaching for certain objects. Alternatively, additional sensors (e.g., cameras and force sensors) could be added to a prosthesis to provide context about the successful grasp or accidental release of an object.

For general human-computer interaction using myoelectric control, the greater availability of contextual information in digital environments could help to develop rich and intuitive control. Each successful click of a button, or the detection of a click when no button is available, could provide context with which to inform model adaptation. Quick corrective actions, such as playing and immediately pausing a music application or passing over then quickly returning to a selection menu, could provide the context of an inadvertent command. By assigning appropriate labels, the system could iteratively adapt and reduce similar classification errors from arising for similar user inputs.

Recognizing that the quality of context engineering impacts the effectiveness of CIIL, the instrumentation does not need to be ubiquitous. Even when only partial access to contextual information is available, such as within a user’s home or during the introduction of new control actions as part of a curriculum, substantial benefits can still be gained. For example, a prosthesis could be trained using CIIL in a rehabilitation environment where context is easy to extract (e.g. using instrumented environments or tests). Furthermore, while CIIL assumes consistent goal-driven behavior from users, it acknowledges the real-world complexity where users may not always adhere to this paradigm. For instance, naturally-limbed individuals gesticulate while talking; however, the context for this scenario is foreseeably unobtainable and as a result CIIL would likely adapt the underlying activity to no motion. As such, there is future work in exploring how to benefit from partial contextual feedback and how to not altogether remove creative expression.

### Future work

This work provides a foundational basis for CIIL approaches that maintain the accuracy of a classifier given a contextual environment; however, there still remain numerous avenues of research to explore and improve the capabilities of this approach.

Future work should investigate the efficacy of CIIL within more realistic, physical environments. Although the instrumentation of these settings is not trivial (vision based, NFC tags, etc.), integrating within existing smart home technologies or commonly used items could prove valuable for all populations that use myoelectric control (general HCI, stroke, spinal cord injury, muscular distrophy, amputees, etc.).

Although this work demonstrated robustness to two confounding factors (minimal training data and electrode shift), there are many other sources of variability that could be minimized with this incremental learning approach (limb position variability, contraction intensity variability, hands-busy interference, between-session differences, cross-subject differences). Electrode shift degradation is typically addressed with recalibration or by making representations of multiple electrode configurations [57]. However, this work demonstrated that an online incremental learning approach could offer a viable solution, adapting to the current electrode position rather than attempting to describe multiple positions.

Within this experiment, a virtual environment was used that had perfect situational context available for every myoelectric decision; however, this may not be the case for real-world systems, such as one relying on object detection for contextual information. In such a scenario, it would be vital to understand the relationship between the accuracy of contextual information and the trajectory of the CIIL training process. Future work should evaluate this relationship to understand the lower-bounds of context accuracy and frequency required for CIIL systems to reliably improve myoelectric usability.

CIIL demonstrated it could improve the classifier’s predictions over time given contextual suitability; however, it could be beneficial to adopt this approach within regression problems—as these are also prevalent in velocity-based myoelectric control applications. Similarly, CIIL should be extended beyond 2-DOF problems to determine if ambiguity among the potential options greatly increases the time required to reach a usable model. While this work demonstrated that CIIL is an effective framework for classification-based online learning that is invariant to initial model performance, future work could leverage structured Bayesian models [58], auto-regressive or nonlinear regression approaches [59, 60], or deep learning to extend its benefits to other tasks such as regression.

Perhaps most importantly, we hope to reduce the complexity involved in implementing CIIL approaches to facilitate the uptake of these methods. This work required constant communication between different processes performing user-in-the-loop classification, collecting the associated context, and periodically performing adaptation. We aim to provide the supporting infrastructure for this style of experiment in LibEMG in the future [44].

## Conclusion

In this paper we presented Context-Informed Incremental Learning (CIIL), a new incremental learning framework for myoelectric control. In two online studies, CIIL was shown to significantly outperform the state-of-the-art approach (unsupervised high-confidence adaptation) when beginning with a sparsely defined classifier trained with minimal data and when recovering from a catastrophic change in the input space due to electrode shift. Additionally, through the adoption of CIIL strategies, myoelectric control systems better incorporate user-in-the-loop behaviours which will in turn improve the ability of models to decipher user intent, resulting in better overall control.

## Data Availability

A link to the analysis code and data is provided within the manuscript (https://github.com/ECEEvanCampbell/CIIL_LDA).

## Footnotes

1 https://coaptengineering.com.

2 https://www.i-biomed.com/.

3 https://www.ottobock.com/.
